# OCT-4 expression in follicular and luteal phase endometrium: a pilot study

**DOI:** 10.1186/1477-7827-8-38

**Published:** 2010-04-22

**Authors:** Eva-Katrin Bentz, Marina Kenning, Christian Schneeberger, Andrea Kolbus, Johannes C Huber, Lukas A Hefler, Clemens B Tempfer

**Affiliations:** 1 Department of Gynecological Endocrinology and Reproductive Medicine, Medical University of Vienna, Vienna, Austria; 2 Department of Obstetrics and Gynecology, Medical University of Vienna, Vienna, Austria

## Abstract

**Background:**

The stem cell marker Octamer-4 (OCT-4) is expressed in human endometrium. Menstrual cycle-dependency of OCT-4 expression has not been investigated to date.

**Methods:**

In a prospective, single center cohort study of 98 women undergoing hysteroscopy during the follicular (n = 49) and the luteal (n = 40) phases of the menstrual cycle, we obtained endometrial samples. Specimens were investigated for OCT-4 expression on the mRNA and protein levels using reverse transcriptase polymerase chain reaction (RT-PCR) and immunohistochemistry. Expression of OCT-4 was correlated to menstrual cycle phase.

**Results:**

Of 89 women sampled, 49 were in the follicular phase and 40 were in the luteal phase. OCT-4 mRNA was detected in all samples. Increased OCT-4 mRNA levels in the follicular and luteal phases was found in 35/49 (71%) and 27/40 (68%) of women, respectively (p = 0.9). Increased expression of OCT-4 protein was identified in 56/89 (63%) samples. Increased expression of OCT-4 protein in the follicular and luteal phases was found in 33/49 (67%) and 23/40 (58%) of women, respectively (p = 0.5).

**Conclusions:**

On the mRNA and protein levels, OCT-4 is not differentially expressed during the menstrual cycle. Endometrial OCT-4 is not involved in or modulated by hormone-induced cyclical changes of the endometrium.

## Background

Octamer-4 (OCT-4) is a homeodomain transcription factor of the Pit-Oct-Unc transcription factor family [1-3]. A transcription factor is defined as a protein binding to specific DNA binding domains and subsequently regulating the transcription from DNA to RNA by activation or repression of RNA polymerase [4]. Specifically, OCT-4 regulates tissue- and cell-specific transcription via the consensus motif ATGCAAAT and its expression is restricted to pluripotent cells. Loss of OCT-4 expression may be associated with the loss of pluripotentiality [5]. During embryogenesis, OCT-4 is initially active as a maternal factor in the oocyte and remains active in embryos throughout the preimplantation period. OCT-4 is involved in the self-renewal of undifferentiated embryonic stem cells and is therefore used as a marker of embryonic stem cells [5-8].

OCT-4 has also been found to be expressed in malignant tissues such as germ cell tumors, embryonic carcinoma cells [9]. Furthermore, breast cancer cells express OCT-4 [10] and OCT-4 is - among other embryonic gene products - re-expressed in cancer cells [11]. Based on these data and the fact that stem cells are undifferentiated, immortal, and invasive, an etiologic role for stem cells as clonogenic origin of various forms of cancer has been proposed [11,12].

Cho et al. described the presence of stem cells in the stroma of the basal layer of human endometrium based on the presence of C-kit/CD 117, CD34, bcl-2, and Ki67 [13]. In a previous study, we demonstrated that OCT-4 is also expressed in the human endometrium, lending further support to the hypothesis of endometrial regeneration by local stem cells in endometrial tissue [12]. This concept is also supported by the presence of clonogenic epithelial and stromal cells in human endometrium acting as putative stem cells [14,15]. It has been speculated that local tissue-specific stem cells are involved in the regeneration and maintenance of the endometrial lining during the follicular phase and the menstruation. This concept, however, has been challenged by evidence that OCT-4 genetic ablation did not result in abnormalities in homeostasis and regenerative capacity in rodent studies [16].

To further investigate the role of OCT-4 in human endometrial physiology, we performed a prospective study to assess the mRNA and protein expression of OCT-4 in follicular and luteal phase endometrium. We hypothesized that OCT-4 is differentially regulated in the follicular and luteal phases of the menstrual cycle. Specifically, we aimed to answer the question whether or not OCT-4 is overexpressed during endometrial proliferation in the follicular phase and downregulated during the secretory transformation of the endometrium in the luteal phase.

## Methods

### Patients

We performed a prospective, single center cohort study between September 2006 and March 2007 in a population of 89 consecutive patients undergoing hysteroscopy and endometrial sampling at the Endoscopy Unit of the Department of Gynecologic Endocrinology and Reproductive Medicine at Vienna Medical University, Vienna, Austria. The mean age of the patients was 33.9 ± 5.2 years. All women had regular menstrual cycles during the last 6 months (menstrual cycle length 25-35 days) and did not take any hormone therapy. Menstrual phase assessment was based on the date of the last menstrual period with the luteal and follicular phases determined by halfing the median number of cycle days of the last three menstrual cycles of the patient and confirmed by histopathological analysis of the endometrial specimen. Indications for surgery were primary sterility (n = 32), secondary sterility (n = 34), endometriosis and/or dysmenorrhea (n = 10), recurrent pregnancy loss (n = 4), chronic pelvic pain (n = 4), and others (n = 5). Written informed consent was obtained by all patients.

### Reverse transcriptase polymerase chain reaction (RT-PCR)

**Figure 1:**
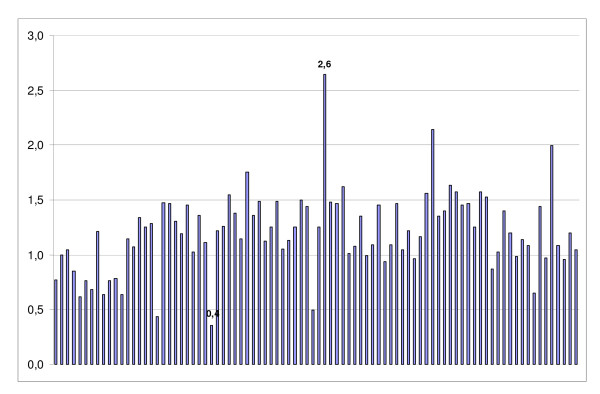
**OCT-4 cDNA expression after RT-PCR relative to GADPH in 89 patients**. OCT-4 = Octamer-4; cDNA = complementary deoxyribonucleic acid; RT-PCR = reverse transcriptase polymerase chain reaction; GADPH = glyceraldehyde-3-phosphatedehydrogenase. Each bar represents one patient; values are given for each patient representing OCT-4 cDNA expression relative to the expression of GADPH with 1 denominating equivalent expression. The lowest (0.4) and highest (2.6) values are shown.

For RNA extraction frozen tissue samples were triturated and total RNA was extracted using the TRI REAGENT method (Molecular Research Centre, Inc., OH, USA). RNA concentration was determined by measuring the optical density at 260 nm. 1 μg RNA was reversely transcribed into first strand complementary DNA (cDNA) using Superscript (Invitrogen Ltd., Paisley, UK). The resulting cDNA was amplified by polymerase chain reaction (PCR) using primers specific for OCT-4 [11]. The following primers were used for RT-PCR reactions: OCT 4 forward 5'-GAC AAC AAT GAA AAT CTT CAG GAG A-3' and OCT reverse 5'-TTC TGG CGC CGG TTA CAG AAC CA-3'. The PCR was started with a denaturing step at 94°C for 5 min and the amplification of the 218 bp product was performed for 35 cycles at 94°C for 30 sec, 61°C for 30 sec and 72°C for 30 sec, and a final extension at 72°C for 10 min. As control for genomic DNA we used extracted RNA only (no cDNA). As positive control for the expression of OCT -4 we used RNA from embryonic carcinoma of testis. Human glyceraldehyd-3-phosphate dehydrogenase (GAPDH) was amplified in parallel reactions as a housekeeping reference gene serving as an internal control for the quantity and quality of the cDNA. Primers for GAPDH were: forward 5'-TCT GGT AAA GTG GAT ATT GTT G-3' and reverse 5'-GAT GGT GAT GGG ATT TCC-3'. The amplified product for GAPDH has a size of 156 base pairs. Amplified samples were separated on 1.5% agarose gels in the presence of ethidium bromide and visualized by the Eagle Eye System (Stratagene, Amsterdam, The Netherlands). Quantitative analysis was performed by densitometric scanning of the gels using the AlphaDigiDoc 1000 software (Alpha Innotech Corp., San Leonardo, CA). OCT-4 mRNA levels were determined by calculating the OCT-4/GADPH ratios (Figure 1). We optimized all PCR steps with respect to sensitivity, reproducibility, and linearity: different template, enzyme, and primer concentrations, reaction times, and temperatures were tested. The resulting optimal conditions are given above. Since the technician and the scoring person were both blinded to the menstrual phase of the specific samples, the samples have been placed in the order of sampling and not arranged according to menstrual phase.

**Figure 2:**
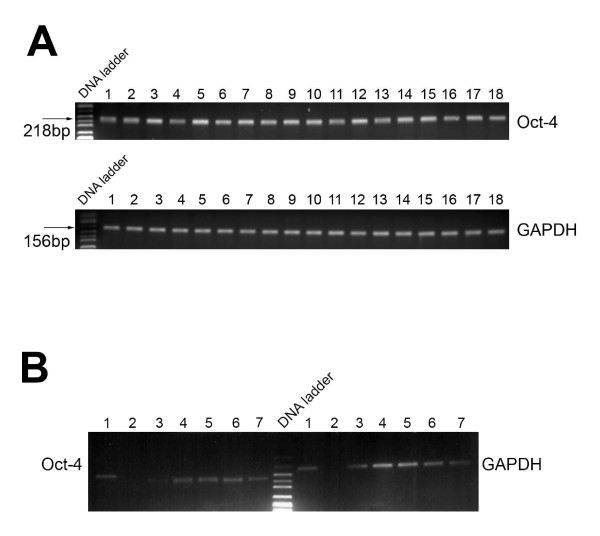
**OCT-4 cDNA expression in endometrial samples using RT-PCR**. OCT-4 = Octamer-4; cDNA = complementary deoxyribonucleic acid; RT-PCR = reverse transcriptase polymerase chain reaction; GADPH = glyceraldehyde-3-phosphatedehydrogenase. (**A**) lanes 1,2,5,6,7,8,10,11,12, and 16: patient samples from the luteal phase; lanes 3,4,9,13,14,15,17, and 18: patient samples from the follicular phase. (**B**) lane 1 = positive control (embryonic carcinoma); lane 2 = negative control (water); lanes 3,4, and 7: patient samples from the luteal phase: lanes 5 and 6: patient samples from the follicular phase.

Although the mRNA quantities varied, expression of OCT-4 was detected in all samples analyzed (Figure 2). By including control reactions with input RNA instead of cDNA obtained after reverse transcription of RNA, we are confident that the OCT-4 PCR product of the endometrial samples is not due to genomic DNA contamination.

Increased mRNA levels were evaluated relative to GAPDH, ie mRNA levels equivalent or less than GAPDH were considered normal, mRNA levels above the levels measured for GAPDH were considered indicative of increased transcription.

For confirmation of OCT-4 expression in endometrial tissue, we performed Western Blot experiments, but were not able to consistently demonstrate OCT-4 protein in the frozen tissue samples used (see Additional File 1, Figure S1).

### Immunohistochemistry

**Figure 3:**
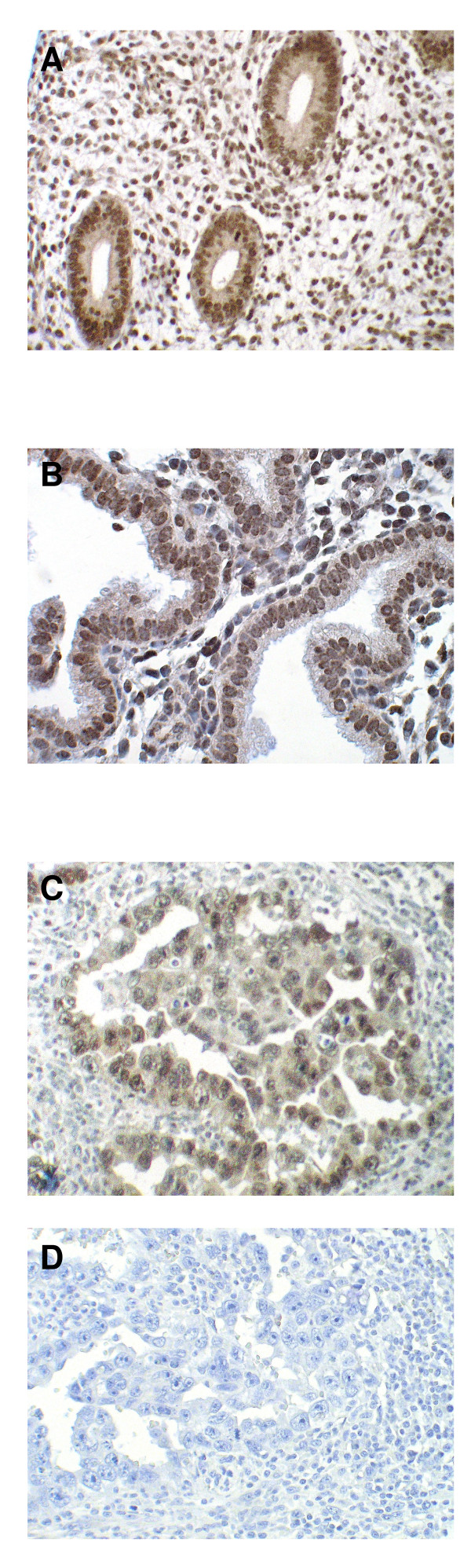
**Immunohistochemical staining for OCT-4**. OCT-4 = Octamer-4; (**A**) OCT-4 overexpression in a sample of follicular phase endometrium. The picture demonstrates selected tissue compartments, ie three endometrial glands and surrounding endometrial stroma The brown staining shows OCT-4 expression in the cytosol of glandular cells. Magnification ×100. (**B**) OCT-4 overexpression in a sample of luteal phase endometrium. The picture demonstrates selected tissue compartments, ie two secretory endometrial glands and surrounding endometrial stroma The brown staining shows OCT-4 expression in the cytosol of glandular cells. Magnification ×200. (**C**) OCT-4 overexpression in a sample of embryonic carcinoma of the testis serving as positive control. Magnification ×200. (**D**) Section of embryonic carcinoma of the testis stained with IgG1 as described in Materials and Methods serving as negative control. Magnification ×200.

We used formalin-fixed, paraffin-embedded tissue samples of endometrium from 89 patients. From these sections we performed immunohistochemical staining using a primary antibody recognizing OCT-4 (OCT-4 [C-10]: sc-5279, Santa Cruz Biotechnology, California) as previously described [12]. Stained sections were scored according to the immunoreactive score (IRS) described by Remmele and Stegner [17]. The IRS results from the multiplication of a staining intensity score (negative = 0; weak = 1; moderate = 2; strong = 3) and the percentage score of immunopositive cells (no staining = 0, 1-10% of stained cells = 1; 11-50% of stained cells = 2, 51-80% of stained cells = 3; 81-100% of stained cells = 4). Each sample was scored according to these IRS criteria. An IRS of ≥6 was considered to indicate 'increased expression' of OCT-4. Figures 3A, B, C, and 3D show examples with a strong (A, B, C) and an absent (D) staining intensity, respectively.

### Statistics

Categorical variables were analyzed by *x*^2^-test. Multiple comparisons were corrected using Bonferroni's correction. OCT-4 mRNA and protein expression were correlated using Pearson's correlation coefficient. A univariate regression model was used to assess the influence of day of the menstrual cycle, using 3-day intervals, on OCT-4 protein expression, comparing an IRS <6 vs. ≥ 6. All p-values were two-tailed and 95% CI were calculated. A p-value < 0.05 was considered statistically significant. We used the statistical software SPSS 11.0 for Windows (SPSS Inc., Chicago, IL) for statistical analyses.

## Results

We included 89 patient samples. The mean age of the patients was 33.9 ± 5.2 years. Of these, 49 were in the follicular phase and 40 were in the luteal phase. All patients underwent hysteroscopy and endometrial sampling. Indications for surgery were primary sterility (n = 32), secondary sterility (n = 34), endometriosis and/or dysmenorrhea (n = 10), recurrent pregnancy loss (n = 4), chronic pelvic pain (n = 4), others (n = 5). OCT-4 mRNA was detected in all samples (Figure 1). Increased OCT-4 mRNA levels were observed in 62/89 (70%) samples. Comparing the number of samples with increased OCT-4 mRNA levels in the follicular and luteal phases, we found no statistically significant difference (35/49 [71%] vs. 27/40 [68%], respectively (p = 0.9). Figure 2 demonstrates OCT-4 cDNA expression in endometrial samples of the follicular and luteal phases.

OCT-4 protein overexpression was identified in 56/89 (63%) samples. OCT-4 protein overexpression in the follicular and luteal phase was found in 33/49 (67%) and 23/40 (58%) of women, respectively (p = 0.5). In a univariate regression model, the day of the menstrual cycle, using 3-day intervals, did not influence OCT-4 protein expression, when comparing an IRS <6 vs. ≥ 6 (p = n.s). Increased mRNA levels and increased OCT-4 protein expression were significantly correlated (Pearson's correlation coefficient 0.8). Figure 3 shows follicular and luteal phase endometrial samples with and without OCT-4 overexpression. We found that OCT-4 expression was restricted to the plasma of epithelial endometrium cells. This pattern of expression was present in the follicular and luteal phases of the menstrual cycle with no discernible differences.

## Discussion

In the present study we assessed the mRNA levels and protein expression of OCT-4 in human endometrium. We found that human endometrium contains potentially pluripotent OCT-4-expressing cells with increased OCT-4 mRNA levels observed in 70% of samples. OCT-4, however, is not differentially expressed on the mRNA and protein levels during the menstrual cycle. We conclude that human endometrium may contain pluripotent cells. The stem cell marker OCT-4 is widely expressed in human endometrium, but it is not involved in or modulated by hormone-dependent cyclical changes of the endometrium.

Our results compare with other studies in that OCT-4 has been demonstrated to be expressed in the human endometrium [12]. From a functional perspective, Taylor et al. demonstrated donor-derived endometrial cells in bone-marrow transplantation recipients indicating a functional interaction of potential uterine and nonuterine stem cells [14]. It has to be acknowledged, however, that there is not enough evidence available at this time, including our own results, to convincingly conclude that OCT-4 positive cells in the endometrium are pluripotent.

We found that OCT-4 expression was restricted to the cytosol of epithelial endometrium cells. This pattern of expression was present in the follicular and luteal phases of the menstrual cycle with no discernible differences. This rejects our study hypothesis that OCT-4 is overexpressed during endometrial proliferation in the follicular phase and downregulated during the secretory transformation of the endometrium in the luteal phase. This finding does not support the concept of cyclical endometrial renewal based on hormonally modulated local OCT-4 expressing cells. Rather, it supports the notion of a constant and sex steroid-independent pool of renewable stem cells from which cells may be recruited.

Our study has limitations. For example, the semiquantitative technique of immunohistochemistry and the small sample size may miss subtle differences in OCT-4 expression. Thus, we can only rule out a profound influence of menstrual cycle-dependent changes of the human endometrium on OCT-4 expression. Also, OCT-4 is a surrogate marker of stem cell potential rather than a direct proof of pluripotency. Thus, other yet unknown factors influencing the expression of OCT-4 may be present in human endometrium.

Regarding the implications of our results for future research, we suggest that the investigation of peri- and postmenopausal endometrium may be worthwile. Such experiments may determine whether or not endometrial expression of OCT-4 - as demonstrated in this study - will decrease at the end of the reproductive period. Also, differential regulation of OCT-4 expression in women with endometriosis may be a worthwile target of investigation.

In conclusion, we evaluated the mRNA-transcription and protein expression of the stem cell marker OCT-4 in human endometrium and found that OCT-4 is frequently expressed in human endometrium, but is not involved in menstrual cycle regulation.

## Conclusions

On the mRNA and protein levels, OCT-4 is not differentially expressed during the menstrual cycle. Endometrial OCT-4 is not involved in or modulated by hormone-induced cyclical changes of the endometrium.

## Competing interests

The authors declare that they have no competing interests.

## Authors' contributions

EKB conceived the study and drafted the manuscript. CS and AK carried out the molecular and immunohistochemical experiments. MK participated in the immunohistochemical experiments. JCH participated in the design and coordination of the study. LAH performed the statistical manuscript and helped to draft the manuscript. CBT participated in the design and coordination of the study and drafted the manuscript. All authors read and approved the final manuscript.

## Supplementary Material

Additional file 1 Supplemental Figure 1: OCT_4_Western_Blots Click here for file
