# Supplementary figures and images for: OCT-4 expression in follicular and luteal phase endometrium: a pilot study
Source: Reprod Biol Endocrinol. 2010 Apr 22;8:38. doi: 10.1186/1477-7827-8-38 (PMC2867815; doi:10.1186/1477-7827-8-38)

## Oct-4 – Western Blot Project - Figure “WB results”

Gel 1:

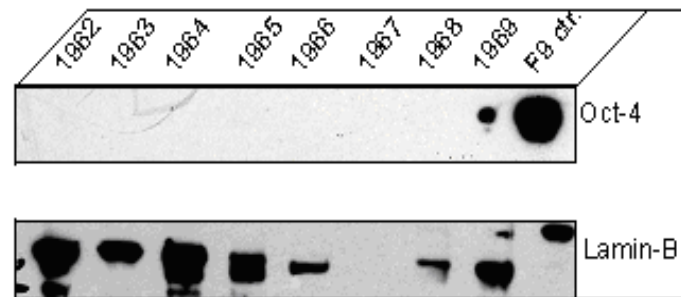

Gel 2

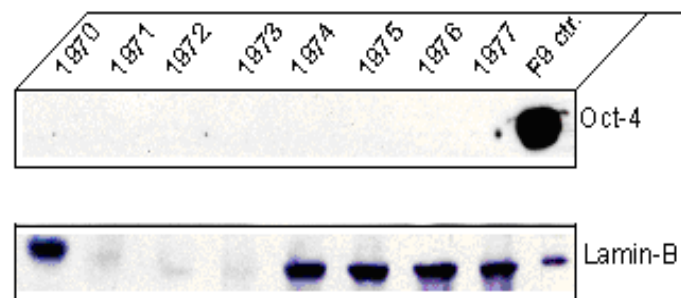

Gel 3

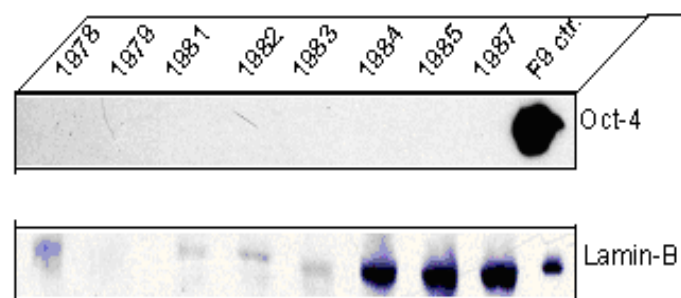

Gel 4

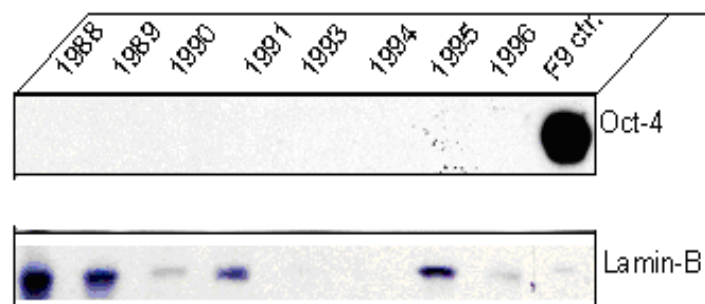

Gel 5

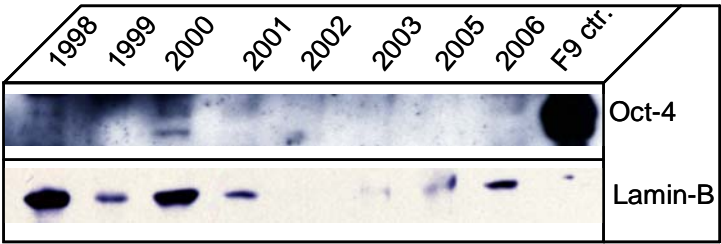

Gel 6

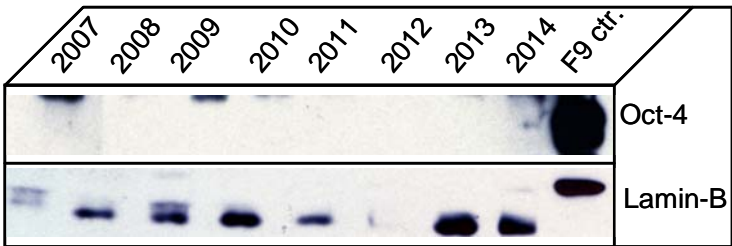

Gel 7

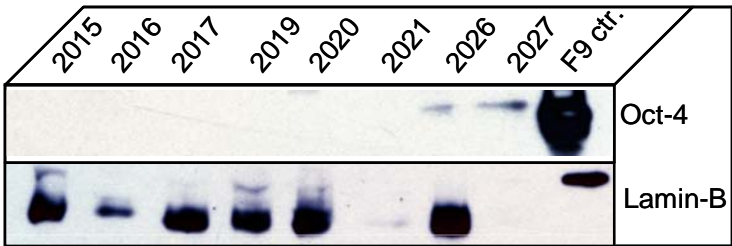

Gel 8

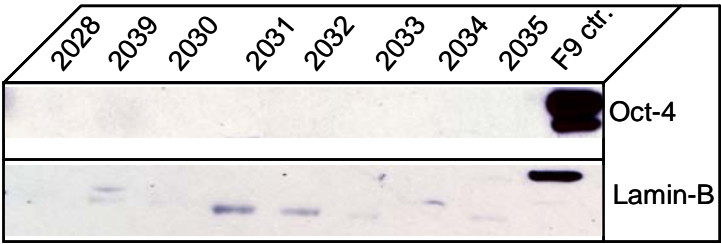

Gel 9

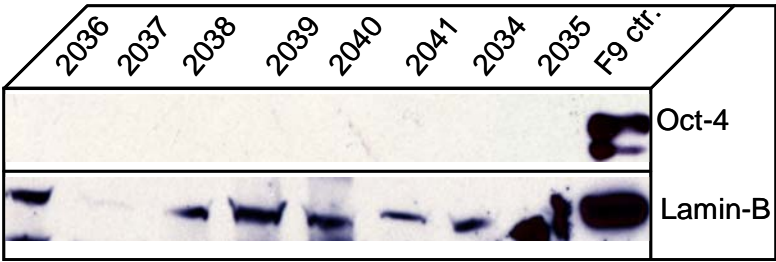

Gel 10

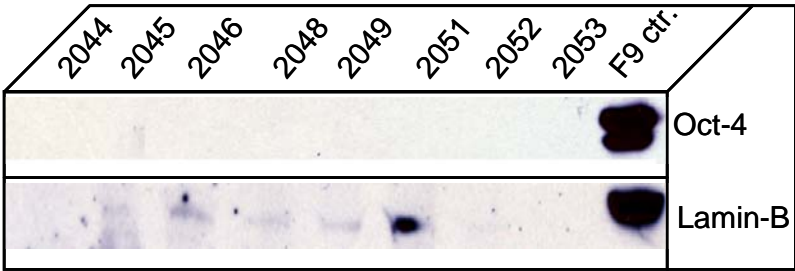

Supplement: Additional file 1 — Supplemental Figure 1: OCT_4_Western_Blots [file 1477-7827-8-38-S1.PDF]
